# Association of intensive blood pressure management with cardiovascular outcomes in patients using multiple classes of antihypertensive medications: a post-hoc analysis of the STEP Trial

**DOI:** 10.1038/s41440-024-01647-1

**Published:** 2024-04-10

**Authors:** Kaipeng Zhang, Qirui Song, Jingjing Bai, Jun Cai

**Affiliations:** 1 4 + 4 Medical Doctor Program, Chinese Academy of Medical Sciences & Peking Union Medical College, No.9 Dongdansantiao Street, Dongcheng District, Beijing, 100730 China; 2 Hypertension Center, Fuwai Hospital, State Key Laboratory of Cardiovascular Disease of China, National Center for Cardiovascular Diseases of China, Key Laboratory of Cardiometabolic Diseases, Chinese Academy of Medical Sciences and Peking Union Medical College, Beilishi Rd. 167, Xicheng District Beijing, 100037 China

**Keywords:** Hypertension, Intensive blood pressure management, Medication burden, Cardiovascular outcomes

## Abstract

High medication burden is associated with poor treatment effect and high risk of cardiovascular outcomes. This study aimed to investigate the association between the antihypertensive medication burden and cardiovascular outcomes in the STEP trial. This post-hoc analysis of the STEP trial enrolled 8511 participants, including 8041 with low burden and 470 with high burden. High antihypertensive medication burden was defined as being treated with ≥3 different classes of prescribed antihypertensive medications. The primary outcome was a composite of cardiovascular outcomes. Fine-Gray model was used in this study. Among all participants, high antihypertensive medication burden was associated with a higher risk of the primary outcome compared with low medication burden (HR, 1.52; 95% CI, 1.03–2.24), which was consistent in the standard group (HR, 1.95; 95% CI, 1.20–3.18) and the intensive group (HR, 1.10; 95% CI, 0.57–2.13; *P*_interaction_ = 0.18). The beneficial effects of intensive systolic blood pressure (SBP) control on the primary outcome remained significant in the high burden group (HR, 0.42; 95% CI, 0.19–0.95) and the low burden group (HR, 0.79; 95% CI, 0.63–0.98; *P*_interaction_ = 0.18). At 24 months, the percentage of participants achieving the target SBP was lower in the high medication burden group (risk ratio, 0.93; 95% CI, 0.89–0.98). In both standard and intensive treatment groups, participants with a high medication burden were harder to achieve the target SBP (*P*_interaction_ = 0.65). High antihypertensive medication burden was associated with worse SBP control and a greater risk of cardiovascular events. Intensive SBP control showed cardiovascular benefits in both medication burden groups. Trial registration: STEP ClinicalTrials.gov number, NCT03015311. Registered 2 January 2017.

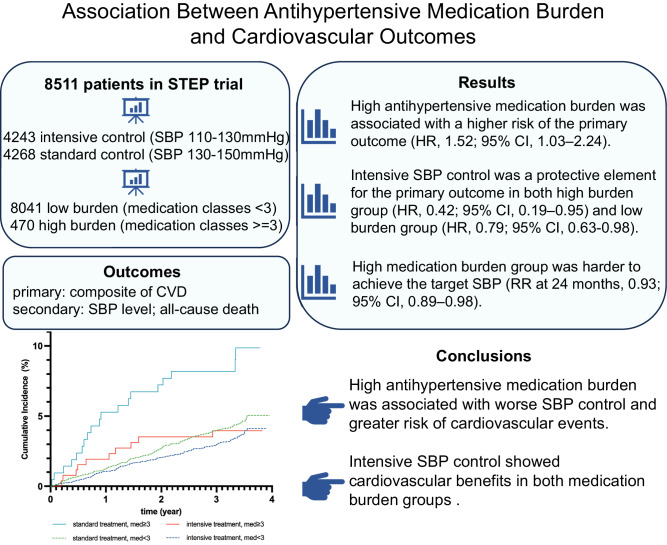

## Introduction

Along with the continuous development of society and the conspicuous improvement of living standard, the incidence of chronic non-communicable diseases such as hypertension, hyperlipidemia and diabetes are increasing. The prevalence of hypertension was 34.0% worldwide in 2020 [1], and was 29.8% in China in 2018 [2]. Due to the increasing hypertension population and the close association between hypertension and cardiovascular events, elevated systolic blood pressure (SBP) has become the most important risk factor for cardiovascular disease (CVD) worldwide [3–5]. A 10 mmHg reduction of SBP could significantly reduce the risk of major cardiovascular events by about 20%, and the reduction of SBP could provide protection for coronary heart disease, stroke, heart failure and many other diseases [6, 7].

To reduce the risk of elevated SBP, recent hypertension guidelines have proposed various treatment recommendations. However, the control rate of hypertension is still incredibly low. The factors leading to unsatisfactory SBP control included the need for multiple medications, complexity of medical dosages, interaction and adverse effects of different medications, therapeutic inertia and so on [8, 9]. Low treatment adherence has also been considered as a pivotal factor, which might be associated with medication burden, age, comorbid diseases and other factors [10–12]. Therefore, high medication burden may contribute to poor SBP control. Several studies have also expounded that high medication burden could lead to higher risk of cardiovascular events and worse self-rated health in particular crowds [13, 14].

Recently, the Systolic Blood Pressure Intervention Trial (SPRINT) researched the association between baseline medication burden and CVD events [15]. However, the association between total antihypertensive medication burden and cardiovascular events in Chinese elder hypertension patients is still under investigation. Therefore, this study aimed to analyze the results of the Strategy of Blood Pressure Intervention in the Elderly Hypertensive Patients (STEP) trial to demonstrate the association between the antihypertensive medication burden and the SBP control, CVD events, all-cause mortality, and medication adherence in the Chinese population. We supposed that high antihypertensive medication burden would be associated with worse SBP control, higher risk of CVD events and worse medication adherence. We also wanted to gather more evidence of intensive SBP control’s cardiovascular benefits to guide clinical treatment.

Point of view

- Clinical relevance:
  High antihypertensive medication burden was associated with adverse cardiovascular outcomes, whereas intensive SBP control demonstrated cardiovascular benefits in both medication burden groups.
- Future direction:
  Future analyses should investigate whether high antihypertensive medication burden remains associated with a greater risk of cardiovascular events when considering the influence of inherent confounding factors.
- Consideration for the Asian population:
  The use of intensive antihypertensive therapy and the number of antihypertensive medications administered remains controversial in Asia.

## Methods

### Study design and participants

The rationale and design of the STEP trial, which was a prospective, multicenter, randomized, controlled trial performed at 42 clinical centers throughout China, have been published previously [16, 17]. Briefly, the STEP trial compared the effects of an intensive SBP target of 110–130 mmHg versus a standard target of 130–150 mmHg on cardiovascular outcomes. The STEP trial included patients aged 60–80 years who had an SBP of 140–190 mmHg or were receiving antihypertensive medication. Participants with prior ischemic or hemorrhagic stroke were excluded. From 10th January 2017 to 31st December 2017, 8511 patients were screened, recruited, and randomly divided into an intensive treatment group of 4243 participants and a standard treatment group of 4268 participants. The STEP trial was approved by the Ethics Committee of Fuwai Hospital and collaborating centers. All participants provided written informed consent.

### Randomization, intervention, and follow-up

A central computer program accessed via a website was used to randomly divide all eligible participants into the intensive treatment group or the standard treatment group in a 1:1 ratio. The participants were stratified by clinical center. All participants were followed up at specific timepoints after randomization. Participants were screened monthly for the first 3 months, and then every 3 months until the end of the trial or the death of the participant.

### Assessment of medication burden and covariate measurements

We used the information of antihypertensive medications prescribed for participants at randomization to denote overall antihypertensive medication burden, since the prescription was almost the same during the whole follow-up visits for most participants. A high antihypertensive medication burden was defined as treating with three or more different classes of antihypertensive medications, because this number of medications has frequently been used to identify resistant hypertension.

Trained physicians evaluated all participants using a standardized questionnaire during face-to-face visits. The questionnaire collected baseline information, including demographic characteristics (e.g., age, sex, birth date, residence, weight, height), lifestyle behaviors (e.g., physical activity, drinking status, smoking status), medical history (e.g., diabetes mellitus, hyperlipidemia, CVD, other chronic diseases), and medication use (e.g., antihypertensive agents, statins, aspirins). At each visit, the physicians measured the office blood pressure and heart rate, and collected information on concomitant medication use, antihypertensive drug adherence (assessed using the Morisky Medication Adherence Scale-8), and trial outcomes. Patients were required to rest for at least 5 min in a seated position, then a well-trained physician measured the blood pressure three times with an interval of 1 min, and the average value was recorded as the final datum. This process was standardized and all of the office blood-pressure monitors (Omron Healthcare) were same at all participating centers and validated during all clinic visits.

All participants underwent laboratory blood testing at baseline and each year thereafter, and biological examinations were conducted to acquire the total cholesterol, triglyceride, low-density lipoprotein cholesterol (LDL-C), high-density lipoprotein cholesterol, fasting plasma glucose, creatinine, and uric acid concentrations. Body mass index (BMI) was calculated as the weight (kilograms) divided by the squared height (meters). The Chronic Kidney Disease Epidemiology Collaboration formula was used to estimate the glomerular filtration rate. The Framingham risk score was calculated to evaluate the 10-year CVD risk of all participants [18].

### Outcomes

The primary outcome was a composite of stroke (ischemic or hemorrhagic), acute decompensated heart failure, acute coronary syndrome (acute myocardial infarction and hospitalization for unstable angina), atrial fibrillation, coronary revascularization, or death from cardiovascular causes. The secondary outcomes were SBP level and all-cause death within each treatment group. The detailed definitions and ascertainment criteria for the study outcomes have been published [16].

### Statistical analysis

Continuous variables are presented as mean ± standard deviation, while categorical variables are presented as *n* (%). Baseline characteristics were compared across baseline medication burden groups within the intensive and standard treatment groups using one-way analysis of variance for continuous variables and the chi-squared test for categorical variables.

The relationship between the antihypertensive medication burden and cardiovascular outcomes was analyzed in the total cohort including all participants, and in the intensive and standard treatment groups separately. The ordinary least squares regression model was applied to calculate the mean SBP and changes in SBP from baseline to 12, 24, and 36 months. Modified Poisson regression was used to calculate the risk ratios (RRs) for achieving the target SBP. The Fine-Gray model was used to calculate hazard ratios (HRs) for the primary outcome, and Cox proportional hazard regression was used to calculate HRs for all-cause mortality. Models were adjusted for potential confounders including age, sex, BMI, baseline SBP, estimated glomerular filtration rate, fasting plasma blood glucose concentration, LDL-C concentration, smoking frequency, drinking frequency, and physical activity frequency. In addition, we calculated each outcome by including the product term (treatment group × medication burden group) in regression models to detect interactions between the treatment group and medication burden group.

To determine whether the antihypertensive medication burden could modify the effect of intensive versus standard treatment, the Fine-Gray model and Cox proportional hazard regression were used to calculate HRs for the primary outcome and all-cause mortality, respectively, associated with intensive versus standard treatment within the high and low antihypertensive medication burden groups. Furthermore, to investigate whether the results were sensitive to our definition of medication burden, we repeated all analyses with the medication burden groups classified as less than two medications and two or more medications.

All analyses were performed using R version 4.1.2. A two-sided *P* < 0.05 was considered statistically significant.

## Results

### Baseline characteristics

**Fig. 1:**
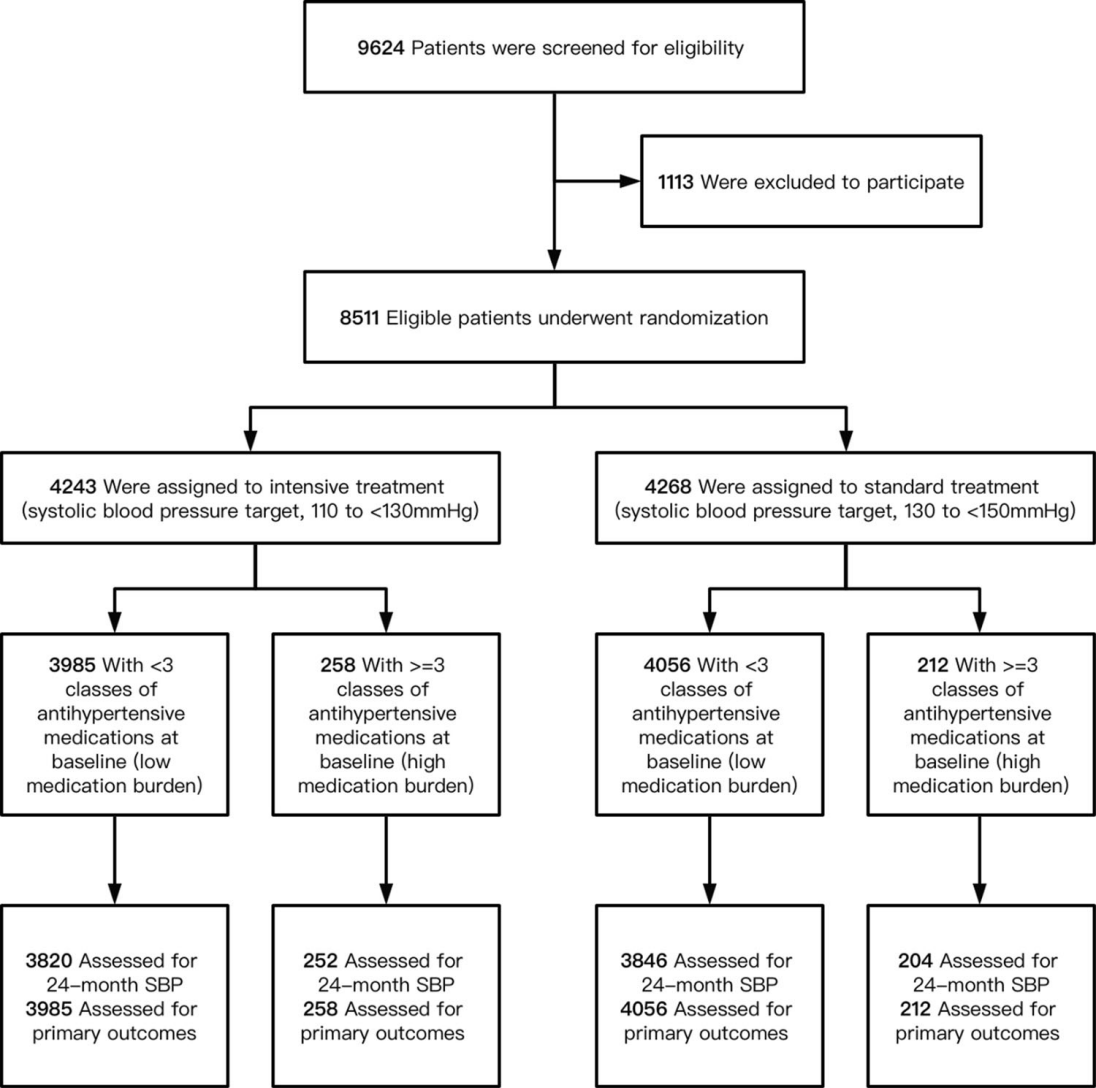
Consolidated standards of reporting trials flow diagram. SBP, systolic blood pressure. Primary outcomes, missing outcomes, and covariates are defined in the Methods section

Among 8511 eligible participants in the STEP trial, 4243 participants were assigned to the intensive treatment group, and 4268 participants were assigned to the standard treatment group. The participant characteristics were shown in Table 1. There were 8041 participants (94.5%) with a low medication burden (<3 classes of antihypertensive medications) and 470 (5.5%) with a high medication burden (≥3 classes of antihypertensive medications) at baseline (Fig. 1). In both the intensive and standard treatment groups, participants with a high medication burden were more likely to be older and to have a higher BMI and higher SBP at baseline than those with a low medication burden. Participants with a high medication burden tended to have lower concentrations of total cholesterol, high-density lipoprotein cholesterol, and LDL-C, and to be more likely to use statins and aspirin. Participants with a high medication burden also tended to smoke and drink more often than those with a low medication burden.

**Table 1:** Baseline characteristics by treatment group and medication burden

Characteristic	Intensive Treatment			Standard Treatment		
	No. of Baseline Medication Classes		*P* Value	No. of Baseline Medication Classes		*P* Value
	<3	≥3		<3	≥3	
Participants, *n*	3985	258		4056	212	
Age, y	66.17 ± 4.83	66.52 ± 5.12	0.27	66.25 ± 4.80	67.19 ± 4.89	0.01
Men (%)	1882 (47.23)	108 (41.86)	0.11	1874 (46.20)	95 (44.81)	0.74
Body mass index, kg/m^2^	25.51 ± 3.15	25.97 ± 3.41	0.02	25.56 ± 3.16	26.55 ± 3.34	<0.001
Systolic blood pressure, mmHg	145.85 ± 16.67	150.72 ± 17.72	<0.001	145.78 ± 16.41	149.64 ± 17.45	<0.001
Diastolic blood pressure, mmHg	82.66 ± 10.60	83.10 ± 11.34	0.52	82.28 ± 10.50	82.12 ± 10.59	0.83
Fasting blood glucose, mmol/L	6.09 ± 1.59	6.04 ± 1.45	0.66	6.15 ± 1.60	6.31 ± 1.63	0.17
TC, mmol/L	4.90 ± 1.10	4.75 ± 1.15	0.03	4.88 ± 1.06	4.82 ± 1.10	0.41
HDL-C, mmol/L	1.27 ± 0.31	1.22 ± 0.30	0.04	1.26 ± 0.30	1.20 ± 0.30	0.004
LDL-C, mmol/L	2.69 ± 0.88	2.56 ± 0.88	0.02	2.70 ± 0.87	2.59 ± 0.89	0.07
CR, mmol/L	73.09 ± 17.97	73.91 ± 17.09	0.48	73.30 ± 18.07	74.55 ± 19.93	0.33
eGFR, mL/(min·1.73 m^2^)	109.84 ± 24.54	106.48 ± 23.83	0.03	109.04 ± 23.97	107.36 ± 26.69	0.32
Smoking status (%)			0.59			0.04
Never	2869 (71.99)	180 (69.77)		2931 (72.26)	143 (67.45)	
Former	472 (11.84)	38 (14.73)		469 (11.56)	37 (17.45)	
Occasionally	107 (2.69)	7 (2.71)		118 (2.91)	3 (1.42)	
Often	537 (13.48)	33 (12.79)		538 (13.26)	29 (13.68)	
Drinking status (%)			0.58			0.35
Never	2728 (68.46)	184 (71.32)		2771 (68.32)	137 (64.62)	
Former	202 (5.07)	14 (5.43)		219 (5.40)	14 (6.60)	
Occasionally	609 (15.28)	38 (14.73)		649 (16.00)	32 (15.09)	
Current	446 (11.19)	22 (8.53)		417 (10.28)	29 (13.68)	
Physical activity (%)	3369 (84.54)	224 (86.82)	0.37	3461 (85.33)	172 (81.13)	0.12
Statin use (%)	748 (18.78)	76 (29.46)	<0.001	749 (18.47)	63 (29.72)	<0.001
Aspirin use (%)	316 (7.93)	43 (16.67)	<0.001	342 (8.43)	29 (13.68)	0.01

*TC* total cholesterol, *HDL-C* high-density lipoprotein cholesterol, *LDL-C* low-density lipoprotein cholesterol, *CR* creatinine, *eGFR* estimated glomerular filtration rate

### Associations between antihypertensive medication burden and SBP control and treatment adherence

At the 24-month follow-up visit, the SBP was significantly higher in participants with a high medication burden than those with a low medication burden within the intensive treatment group (127.97 ± 9.06 mmHg versus 126.19 ± 9.40 mmHg, *P* = 0.003; Table 2) and the standard treatment group (138.82 ± 9.57 mmHg versus 136.03 ± 9.30 mmHg, *P* < 0.001; *P*_interaction_ = 0.27; Table 2). Participants in the intensive treatment group with a high medication burden also experienced a greater SBP change from baseline than those with a low medication burden (−22.61 ± 18.83 mmHg versus −19.78 ± 17.90 mmHg, *P* = 0.02; Table 2). The SBP change from baseline to 24 months did not significantly differ between those two medication burden groups within the standard treatment group (−10.70 ± 18.84 mmHg versus −9.77 ± 17.47 mmHg, *P* = 0.46; *P*_interaction_ = 0.27; Table 2).

**Table 2:** Blood pressure outcomes at 24 months, and clinical outcomes by medication burden among intensive and standard treatment groups

Outcomes	Intensive Treatment			Standard Treatment			*P* interaction
	No. of Baseline Medication Classes		*P* Value or RR/HR (95% CI)	No. of Baseline Medication Classes		*P* Value or RR/HR (95% CI)	
	<3	≥3		<3	≥3		
Participants with SBP at 24 m, *n*	3820	252		3846	204		
SBP, mmHg	126.19 ± 9.40	127.97 ± 9.06	0.003	136.03 ± 9.30	138.82 ± 9.57	<0.001	0.27
SBP change, mmHg	−19.78 ± 17.90	−22.61 ± 18.83	0.02	−9.77 ± 17.47	−10.70 ± 18.84	0.46	0.27
achieved SBP target (%)	2784 (72.88)	166 (65.87)	0.93 (0.91–0.95)	3660(95.16)	184 (90.20)	0.97 (0.93–1.00)	0.65
All participants, *n*	3985	258		4056	212		
Primary outcome (%)	137 (3.44)	10 (3.88)	1.10 (0.57–2.13)	177 (4.36)	19 (8.96)	1.95 (1.20–3.18)	0.18
All cause death (%)	62 (1.56)	5 (1.94)	1.11 (0.45–2.78)	58 (1.43)	6 (2.83)	1.79 (0.77–4.19)	0.50

*P*_interaction_ for treatment randomization × medication burden status

*SBP* systolic blood pressure, *RR* risk ratio, *HR* hazard ratio

Among all participants, the percentage of patients who achieved the target SBP at 24 months was lower in the high medication burden group than the low medication burden group (RR, 0.93; 95% CI, 0.89–0.98; Table S1). Within the intensive treatment group, the high medication burden group was still less likely to achieve the SBP target than the low medication group (RR, 0.93; 95% CI, 0.91–0.95; Table 2), which was similar within the standard treatment group (RR, 0.97; 95% CI, 0.93–1.00; *P*_interaction_ = 0.65; Table 2). The associations between the medication burden and SBP control level at the 12- and 36-months follow-up visits were similar to that at 24 months and in the intensive treatment group, the RR of achieving the target SBP with a high versus a low medication burden progressively decreased (all *P*_interaction_ > 0.2; Tables S1 and S3).

**Fig. 2:**
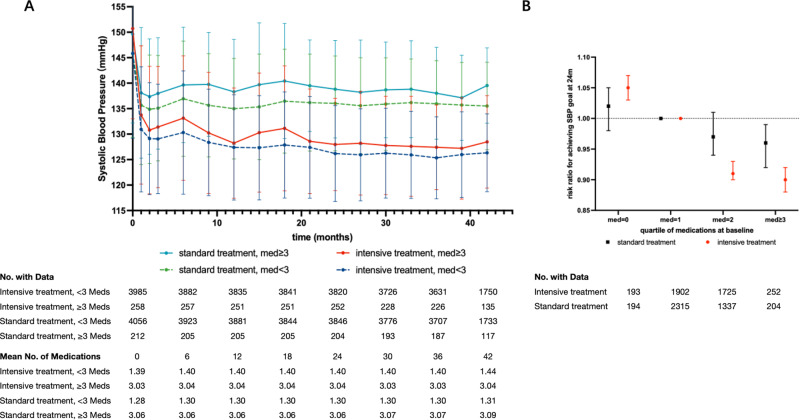
SBP and risk ratios for achieving target SBP by treatment group and medication burden. **A**, Mean SBP throughout the whole STEP trial. **B** Adjusted risk ratios and 95% confidence intervals for achieving the target SBP at 24 months by quartile of baseline medication burden

The mean SBP changes are presented in Fig. 2A. Throughout the whole trial, the mean SBP was higher in the standard treatment group than the intensive treatment group, and the mean SBP of the high medication burden group was higher than that of the low medication burden group within both treatment groups. Furthermore, as the number of medications increased, the probability of achieving the target SBP tended to decrease in both treatment groups at the 12-, 24-, and 36-months follow-up visits (Figs. 2B; S2 and S3).

Throughout the STEP trial, the Morisky Medication Adherence Scale-8 did not significantly differ between the high and low medication burden groups within both treatment groups (all *P*_interaction_ > 0.2; Table S4).

### Association between antihypertensive medication burden and cardiovascular outcomes

**Fig. 3:**
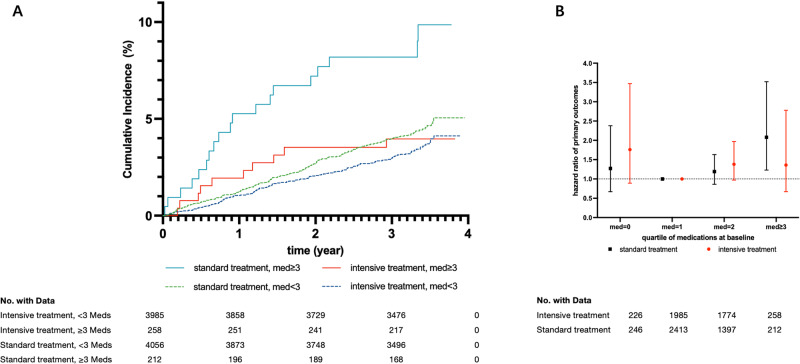
Cumulative hazard plot and hazard ratios for primary outcomes by treatment group and medication burden. **A** Cumulative hazards for primary outcomes throughout the STEP trial. **B** Adjusted risk ratios and 95% confidence intervals for experiencing primary outcomes at the end of the trial by quartile of baseline medication burden

During a median follow-up of 3.34 years, 343 primary outcomes occurred, including 147 in the intensive treatment group and 196 in the standard treatment group. Among all participants, a high medication burden was associated with an increased risk of the primary outcome compared with a low medication burden (HR, 1.52; 95% CI, 1.03–2.24; Table S1). After multivariable adjustment, participants with a high medication burden had a 1.95-fold higher risk of the primary outcome compared with those with a low medication burden in the standard treatment group (HR, 1.95; 95% CI, 1.20–3.18; Table 2). However, the association was not statistically significant in the intensive treatment group (HR, 1.10; 95% CI, 0.57–2.13; Table 2). The effects of medication burden on cardiovascular outcomes were similar within the intensive and standard treatment groups (*P*_interaction _= 0.18; Table 2; Fig. 3A). Furthermore, the primary outcome incidence tended to increase in participants with a high medication burden, regardless of the treatment group (Fig. 3B). The incidence of all-cause mortality did not significantly differ between the high and low medication burden groups in either of the two treatment groups (*P*_interaction _= 0.50; Table 2).

### Effect modification of intensive SBP treatment by antihypertensive medication burden

We also compared the incidence of the primary outcome and all-cause death between the intensive and standard treatment groups after categorizing the participants into high and low medication burden groups. The beneficial effects of intensive versus standard treatment on the primary outcome remained significant in the high medication burden group (HR, 0.42; 95% CI, 0.19–0.95; Table 3) and low medication burden group (HR, 0.79; 95% CI, 0.63–0.98; *P*_interaction_ = 0.18; Table 3), while the effect on all-cause mortality was still insignificant in the high medication burden group (HR, 0.72; 95% CI, 0.21–2.49; Table 3) and low medication burden group (HR, 1.10; 95% CI, 0.77–1.58; *P*_interaction_ = 0.50; Table 3).

**Table 3:** Clinical outcomes by treatment group among high and low medication burden groups

Outcomes	<3 Medication Classes			≥3 Medication Classes			*P* interaction
	Treatment		*P* Value or RR/HR (95% CI)	Treatment		*P* Value or RR/HR (95% CI)	
	Standard	Intensive		Standard	Intensive		
All participants, *n*	4056	3985		212	258		
Primary outcome (%)	177 (4.36)	137 (3.44)	0.79 (0.63–0.98)	19 (8.96)	10 (3.88)	0.42 (0.19–0.95)	0.18
All cause death (%)	58 (1.43)	62 (1.56)	1.10 (0.77–1.58)	6 (2.83)	5 (1.94)	0.72 (0.21–2.49)	0.50

*P*_interaction_ for treatment randomization × medication burden status

*RR* risk ratio, *HR* hazard ratio

### Sensitivity analyses

We conducted sensitivity analyses of all results by re-categorizing the participants into a high medication burden group with two or more medications at baseline and a low medication burden group with less than two medications at baseline. The results of all sensitivity analyses were qualitatively similar to the main analysis (Tables S5–S11; Figs. S4 and S5).

## Discussion

In our study, patients with a high medication burden had a higher risk of cardiovascular events and were less likely to achieve the target SBP compared with those with a low antihypertensive medication burden in both the intensive and standard treatment groups. Moreover, the beneficial effects of intensive versus standard treatment was not changed by the antihypertensive medication burden.

Within both treatment groups, we observed an increase in the SBP and incidence of the primary outcome in the high versus the low medication burden groups throughout the trial. Although the changes were small, such differences in SBP could have tremendous impacts on population health. For instance, lowering the SBP by 2.2 mmHg is predicted to decrease the risks of coronary death and stroke death by approximately 4% and 6%, respectively [19]. We also found that the intensive SBP control showed cardiovascular benefits in both medication burden groups, probably, due to the protective effect of intensive depressurization.

Some studies showed that the more medications that are prescribed, the less likely a patient is to remember or want to take them. The elders with hypertension are more likely to have many comorbid diseases and are therefore often prescribed multiple drugs, which aggravates the aforementioned influence on adherence [20]. In our research, a high medication burden was not associated with significant differences in medication adherence and patient satisfaction during the follow-up period. The medication selection and adherence can be influenced by several patient- and provider-specific factors, such as the cultural background, financial burden, and medication intolerances [21]. Therefore, high medication burden didn’t necessarily lead to low adherence [22, 23]. Moreover, the medication burden in our trial was not as high as that in other studies, and the high management quality of the STEP trial might have contributed to the higher adherence in all groups.

There are no universally acknowledged definitions of high and low medication burdens. A systematic review reported that the most common definition of polypharmacy was five or more medications daily, which was also the definition applied in the SPRINT. Various studies have also applied descriptive definitions or numerical definitions ranging from two to 11 or more medications [15, 24]. Resistant hypertension is defined as an elevated blood pressure above the target despite the concurrent use of three antihypertensive drug classes, or the need for four or more antihypertensive medications to achieve the target blood pressure [25–28]. However, the Chinese population uses relatively fewer medications than the western population, and most participants in the STEP trial were only prescribed one or two drugs; therefore, we chose three classes of antihypertensive medications as the demarcation between high and low medication burdens.

The current study has several limitations. Our adjusted models included extensive clinically important covariates [29]. However, we must consider whether these covariates are truly critical in our analysis and whether other vital covariates need to be included. There could also be some inherent confounding risk, as participants with a higher baseline medication burden are also more likely to have multimorbidity. Furthermore, we could not distinguish medications intended for short-term use or non-antihypertensive use, such as antibiotics and non-steroidal anti-inflammatory drugs, which may have affected our results. Finally, due to the relatively small sample size, especially in the high medication burden groups, we could not analyze the differences in medication burden across race, sex, and comorbidity; these factors warrant investigation in future analyses.

### Perspectives

A high medication burden is becoming increasingly common with the increase in multimorbidity and the aging population. The STEP trial observed the persistent benefits of intensive hypertensive treatment in reducing CVD events. The current study found that a high medication burden was associated with worse SBP control and a higher risk of CVD events in both the standard and intensive treatment groups, and the intensive treatment group showed sustained benefits in both the high and low medication burden groups. The reasons for the lack of an association between the medication burden and patient-reported adherence are unclarified. Further study is warranted to clarify this and identify the factors accounting for our results.

### Asian perspectives

The post-hoc analysis of SPRINT has demonstrated that a high medication burden may increase the risk of cardiovascular events. Nonetheless, it remains uncertain whether these findings are similar in the Asian elderly population due to ethnic variability. Moreover, the utilization of intensive antihypertensive therapy and the number of antihypertensive medications administered remains contentious in Asia. This study provided important insights into the cardiovascular benefits of intensive SBP control and the cardiovascular risk associated with a high antihypertensive medication burden in the Asian elderly population.

## Conclusion

High antihypertensive medication burden was associated with worse SBP control and a greater risk of cardiovascular events. Intensive SBP control showed cardiovascular benefits in both medication burden groups.

### Supplementary information

Supplementary Material
