# Supplementary material for: Association of intensive blood pressure management with cardiovascular outcomes in patients using multiple classes of antihypertensive medications: a post-hoc analysis of the STEP Trial
Source: Hypertens Res. 2024 Apr 10;47(7):1779–88. doi: 10.1038/s41440-024-01647-1 (PMC11224013; doi:10.1038/s41440-024-01647-1)
Supplement: Supplementary file 1 — Supplementary Material [file 41440_2024_1647_MOESM1_ESM.pdf]

Table S1. Blood Pressure Outcomes at 12-, 24- and 36-Months Post-randomization and Clinical Outcomes in All Participants (divided by baseline antihypertensive medication classes < 3 or ≥ 3)

Outcomes	Medications < 3	Medications ≥ 3	RR (95% CI)
Participants with SBP at 12m, n	7716	456	
Achieved SBP target (%)	6351 (82.31)	348 (76.32)	0.95 (0.91-0.99)
Participants with SBP at 24m, n	7666	456	
Achieved SBP target (%)	6444 (84.06)	350 (76.75)	0.93 (0.89-0.98)
Participants with SBP at 36m, n	7338	413	
Achieved SBP target (%)	6461 (88.05)	333 (80.63)	0.93 (0.89-0.97)
All participants, n	8041	470	
Primary outcome (%)	314 (3.90)	29 (6.17)	1.52 (1.03-2.24)
All cause death (%)	120 (1.49)	11 (2.34)	1.43 (0.77-2.66)

SBP, systolic blood pressure; RR, risk ratio.

Table S2. Count of Medications at Baseline and Follow Up Visits by Treatment Group and Baseline Medication Burden (divided by baseline antihypertensive medication classes < 3 or ≥ 3)

Time of Medication Assessment	Intensive treatment		Standard treatment	
	No. of Baseline Medications		No. of Baseline Medications	
	<3	≥3	<3	≥3
Baseline	(N = 3985)	(N = 258)	(N = 4056)	(N = 212)
	1 [1,2]	3 [3,3]	1[1,2]	3 [3,3]
12m Follow Up	(N = 3835)	(N = 251)	(N = 3881)	(N = 205)
	1 [1,2]	3 [3,3]	1[1,2]	3 [3,3]
24m Follow Up	(N = 3820)	(N = 252)	(N = 3846)	(N = 204)
	1 [1,2]	3 [3,3]	1[1,2]	3 [3,3]
36m Follow Up	(N = 3631)	(N = 226)	(N = 3707)	(N = 187)
	1 [1,2]	3 [3,3]	1[1,2]	3 [3,3]

All values are medians and interquartile ranges.

Table S3. Blood Pressure Outcomes at 24- and 36-Months Post-randomization by Treatment Group and Baseline Medication Burden (divided by baseline antihypertensive medication classes < 3 or ≥ 3)

Outcomes	Intensive treatment			Standard treatment			P interaction
	No. of Baseline Medications		P Value or RR/HR (95% CI)	No. of Baseline Medications		P Value or RR/HR (95% CI)	
	<3	≥3		<3	≥3		
Participants with SBP at 24m, n	3820	252		3846	204		
SBP, mm Hg	126.19±9.40	127.97±9.06	0.003	136.03±9.30	138.82±9.57	<0.001	0.27
SBP change, mm Hg	-19.78±17.90	-22.61±18.83	0.02	-9.77±17.47	-10.70±18.84	0.46	0.27
Achieved SBP target (%)	2784 (72.88)	166 (65.87)	0.93 (0.91-0.95)	3660 (95.16)	184 (90.20)	0.97 (0.93-1.00)	0.65
Participants with SBP at 36m, n	3631	226		3707	187		
SBP, mm Hg	125.35±8.05	127.42±8.32	<0.001	135.95±8.47	138.03±9.01	0.001	0.99
SBP change, mm Hg	-20.77±17.93	-23.19±19.36	0.05	-9.95±17.47	-11.15±18.81	0.36	0.50
Achieved SBP target (%)	2856 (78.66)	157 (69.47)	0.90 (0.89-0.92)	3605 (97.25)	176 (94.12)	0.98 (0.95-1.02)	0.42

SBP, systolic blood pressure; RR, risk ratio; HR, hazard ratio.

$P_{interaction}$ for treatment randomization × medication burden status.

Table S4. Patients-reported Adherence at 12-, 24- and 36-Months Post-randomization by Treatment Group and Baseline Medication Burden (divided by baseline antihypertensive medication classes < 3 or ≥ 3)

Outcomes	Intensive treatment			Standard treatment			P interaction
	No. of Baseline Medications		P Value	No. of Baseline Medications		P Value	
	<3	≥3		<3	≥3		
Participants with MMAS at 12m, n	3985	258		4056	212		
high (%)	3421 (85.85)	223 (86.43)	0.99	3468 (85.50)	183 (86.32)	0.33	0.47
moderate (%)	323 (8.11)	21 (8.14)	0.99	307 (7.57)	13 (6.13)	0.47	0.60
low (%)	75 (1.88)	5 (1.94)	0.96	65 (1.60)	2 (0.94)	0.46	0.58
unknown (%)	166 (4.17)	9 (3.49)	0.60	216 (5.33)	14 (6.60)	0.42	0.34
Participants with MMAS at 24m, n	3985	258		4056	212		
high (%)	3433 (86.15)	229 (88.76)	0.45	3522 (86.83)	182 (85.85)	0.39	0.26
moderate (%)	266 (6.68)	18 (6.98)	0.90	210 (5.18)	16 (7.55)	0.14	0.34
low (%)	60 (1.51)	0 (0.00)	0.05	53 (1.31)	1 (0.47)	0.29	0.55
unknown (%)	226 (5.67)	11 (4.26)	0.34	271 (6.68)	13 (2.12)	0.75	0.71
Participants with MMAS at 36m, n	3985	258		4056	212		
high (%)	3384 (84.92)	218 (84.50)	0.03	3460 (85.31)	176 (83.02)	0.29	0.47
moderate (%)	212 (5.32)	5 (1.94)	0.02	195 (4.81)	7 (3.30)	0.38	0.36
low (%)	31 (0.78)	2 (0.78)	0.96	13 (0.32)	0 (0.00)	0.42	0.62
unknown (%)	358 (8.98)	33 (12.79)	0.04	388 (9.57)	29 (13.68)	0.05	0.91

MMAS, Morisky Medication Adherence Scale.

$P_{interaction}$ for treatment randomization × medication burden status.

Table S5. Baseline Characteristics by Treatment Group and Baseline Medication Burden (divided by baseline antihypertensive medication classes < 2 or ≥ 2)

Characteristic	Intensive Treatment			Standard Treatment		
	No. of Baseline Medications		P Value	No. of Baseline Medications		P Value
	<2	≥2		<2	≥2	
Participants, n	2211	2032		2659	1609	
Age, y	66.26±4.88	66.12±4.80	0.33	66.22±4.81	66.41±4.80	0.23
Men (%)	1000 (45.23)	990 (48.72)	0.03	1174 (44.15)	795 (49.41)	<0.001
Body mass index, kg/m^2	25.30±3.15	25.79±3.17	<0.001	25.38±3.11	25.98±3.23	<0.001
Systolic blood pressure, mm Hg	143.65±16.23	148.85±16.93	<0.001	144.07±15.73	149.12±17.21	<0.001
Diastolic blood pressure, mm Hg	82.21±10.50	83.20±10.78	0.002	81.78±10.30	83.09±10.78	<0.001
Fasting blood glucose, mmol/L	5.99±1.49	6.19±1.67	<0.001	6.10±1.57	6.26±1.63	0.003
TC, mmol/L	4.91±1.11	4.86±1.11	0.14	4.88±1.04	4.87±1.09	0.63
HDL-C, mmol/L	1.27±0.31	1.26±0.31	0.17	1.27±0.30	1.25±0.31	0.11
LDL-C, mmol/L	2.71±0.87	2.65±0.88	0.02	2.71±0.85	2.66±0.90	0.06
CR, mmol/L	72.37±17.79	73.98±18.01	0.003	72.70±17.62	74.45±18.99	0.002
eGFR, mL/(min·1.73m^2)	110.33±24.34	108.88±24.68	0.06	109.19±23.61	108.56±24.92	0.40
Smoking status (%)			0.12			<0.001
Never	1615 (73.04)	1434 (70.57)		1984 (74.61)	1090 (67.74)	
Former	250 (11.31)	260 (12.80)		285 (10.72)	221 (13.74)	
Occasionally	65 (2.94)	49 (2.41)		76 (2.86)	45 (2.80)	
Often	281 (12.71)	289 (14.22)		314 (11.81)	253 (15.72)	
Drinking status （%）			0.18			<0.001
Never	1547 (69.97)	1365 (67.18)		1875 (70.52)	1033 (64.20)	
Former	101 (4.57)	115 (5.66)		129 (4.85)	104 (6.46)	
Occasionally	327 (14.79)	320 (15.75)		421 (15.83)	260 (16.16)	
Current	236 (10.67)	232 (11.42)		234 (8.80)	212 (13.18)	
Physical activity（%）	1868 (84.49)	1725 (84.89)	0.75	2268 (85.30)	1365 (84.84)	0.72
Statin use (%)	403 (18.24)	421 (20.72)	0.05	485 (18.24)	327 (20.32)	0.10
Aspirin use (%)	159 (7.19)	200 (9.84)	0.002	224 (8.42)	147 (9.14)	0.46

TC, total cholesterol; HDL-C, high-density lipoprotein cholesterol; LDL-C, low-density lipoprotein cholesterol; CR, creatinine; eGFR, estimated glomerular filtration rate.

$P_{interaction}$ for treatment randomization × medication burden status.

Table S6. Blood Pressure Outcomes at 12-, 24- and 36-Months Post-randomization and Clinical Outcomes in All Participants (divided by baseline antihypertensive medication classes < 2 or ≥ 2)

Outcomes	Medications < 2	Medications ≥ 2	RR (95% CI)
Participants with SBP at 12m, n	4632	3540	
Achieved SBP target (%)	3929 (84.82)	2770 (78.25)	0.94 (0.92-0.96)
Participants with SBP at 24m, n	4604	3518	
Achieved SBP target (%)	4024 (87.40)	2770 (78.74)	0.92 (0.90-0.93)
Participants with SBP at 36m, n	4407	3344	
Achieved SBP target (%)	3984 (90.40)	2810 (84.03)	0.94 (0.92-0.95)
All participants, n	4870	3641	
Primary outcome (%)	173 (3.55)	170 (4.67)	1.25 (1.00-1.55)
All cause death (%)	78 (1.60)	53 (1.46)	0.83 (0.58-1.18)

SBP, systolic blood pressure; RR, risk ratio.

Table S7. Blood Pressure Outcomes at 12-Months Post-randomization and Clinical Outcomes by Treatment Group and Baseline Medication Burden (divided by baseline antihypertensive medication classes < 2 or ≥ 2)

Outcomes	Intensive Treatment			Standard Treatment			P interaction
	No. of Baseline Medications		P Value or RR/HR (95% CI)	No. of Baseline Medications		P Value or RR/HR (95% CI)	
	<2	≥2		<2	≥2		
Participants with SBP at 12m, n	2109	1977		2523	1563		
SBP, mm Hg	126.78±9.76	128.16±10.94	<0.001	134.52±9.74	136.17±10.10	<0.001	0.55
SBP change, mm Hg	-16.88±17.31	-20.77±18.25	<0.001	-9.53±16.71	-12.93±18.31	<0.001	0.54
Achieved SBP target (%)	1528 (72.45)	1314 (66.46)	0.95 (0.93-0.97)	2401 (95.16)	1456 (93.15)	0.99 (0.95-1.03)	0.19
All participants, n	2211	2032		2659	1609		
Primary outcome (%)	66 (2.99)	81 (3.99)	1.28 (0.92-1.78)	107 (4.02)	89 (5.53)	1.29 (0.96-1.72)	0.99
All cause death (%)	40 (1.81)	27 (1.33)	0.67 (0.41-1.11)	38 (1.43)	26 (1.62)	1.00 (0.60-1.67)	0.19

SBP, systolic blood pressure; RR, risk ratio; HR, hazard ratio.

$P_{interaction}$ for treatment randomization × medication burden status.

Table S8. Count of Medications at Baseline and Follow Up Visits by Treatment Group and Baseline Medication Burden (divided by baseline antihypertensive medication classes < 2 or ≥ 2)

Time of Medication Assessment	Intensive treatment		Standard treatment	
	No. of Baseline Medications		No. of Baseline Medications	
	<2	≥2	<2	≥2
Baseline	(N = 2211)	(N = 2032)	(N = 2659)	(N = 1609)
	1 [1,1]	2 [2,2]	1 [1,1]	2 [2,2]
12m Follow Up	(N = 2109)	(N = 1977)	(N = 2523)	(N = 1563)
	1 [1,1]	2 [2,2]	1 [1,1]	2 [2,2]
24m Follow Up	(N = 2095)	(N = 1977)	(N = 2509)	(N = 1541)
	1 [1,1]	2 [2,2]	1 [1,1]	2 [2,2]
36m Follow Up	(N = 1990)	(N = 1867)	(N = 2417)	(N = 1477)
	1 [1,1]	2 [2,2]	1 [1,1]	2 [2,2]

All values are medians and interquartile ranges.

Table S9. Blood Pressure Outcomes at 24- and 36-Months Post-randomization by Treatment Group and Baseline Medication Burden (divided by baseline antihypertensive medication classes < 2 or ≥ 2)

Outcomes	Intensive treatment			Standard treatment			P interaction
	No. of Baseline Medications		P Value or RR/HR (95% CI)	No. of Baseline Medications		P Value or RR/HR (95% CI)	
	<2	≥2		<2	≥2		
Participants with SBP at 24m, n	2095	1977		2509	1541		
SBP, mm Hg	125.30±8.55	127.35±10.11	<0.001	135.47±9.08	137.32±9.64	<0.001	0.64
SBP change, mm Hg	-18.39±17.47	-21.61±18.35	<0.001	-8.64±16.90	-11.74±18.38	<0.001	0.88
Achieved SBP target (%)	1607 (76.71)	1343 (67.93)	0.91 (0.89-0.93)	2417 (96.33)	1427 (92.60)	0.97 (0.94-1.01)	0.09
Participants with SBP at 36m, n	1990	1867		2417	1477		
SBP, mm Hg	124.72±7.58	126.27±8.51	<0.001	135.69±8.31	136.64±8.80	<0.001	0.11
SBP change, mm Hg	-19.09±17.52	-22.86±18.35	<0.001	-8.44±16.93	-12.57±18.22	<0.001	0.66
Achieved SBP target (%)	1617 (81.26)	1396 (74.77)	0.93 (0.91-0.95)	2367 (97.93)	1414 (95.73)	0.99 (0.95-1.02)	0.22

SBP, systolic blood pressure; RR, risk ratio; HR, hazard ratio.

$P_{interaction}$ for treatment randomization × medication burden status.

Table S10. Patients-reported Adherence at 12-, 24- and 36-Months Post-randomization by Treatment Group and Baseline Medication Burden (divided by baseline antihypertensive medication classes < 2 or ≥ 2)

Outcomes	Intensive treatment			Standard treatment			$P_{interaction}$
	No. of Baseline Medications		P Value	No. of Baseline Medications		P Value	
	<2	≥2		<2	≥2		
Participants with MMAS at 12m, n	2211	2032		2659	1609		
high (%)	1876 (84.85)	1768 (87.01)	0.42	2253 (84.73)	1398 (86.89)	0.91	0.63
moderate (%)	182 (8.23)	162 (7.97)	0.64	194 (7.30)	126 (7.83)	0.67	0.53
low (%)	45 (2.04)	35 (1.72)	0.41	46 (1.73)	21 (1.31)	0.24	0.83
unknown (%)	108 (4.88)	67 (3.30)	0.01	166 (6.24)	64 (3.98)	0.001	0.47
Participants with MMAS at 24m, n	2211	2032		2659	1609		
high (%)	1890 (85.48)	1772 (87.20)	0.38	2284 (85.90)	1420 (88.25)	0.37	0.98
moderate (%)	129 (5.83)	155 (7.63)	0.04	132 (4.96)	94 (5.84)	0.33	0.39
low (%)	40 (1.81)	20 (0.98)	0.02	33 (1.24)	21 (1.31)	0.96	0.08
unknown (%)	152 (6.87)	85 (4.18)	<0.001	210 (7.90)	74 (4.60)	<0.001	0.56
Participants with MMAS at 36m, n	2211	2032		2659	1609		
high (%)	1838 (83.13)	1764 (86.81)	0.20	2235 (84.05)	1401 (87.07)	0.18	0.99
moderate (%)	119 (5.38)	98 (4.82)	0.28	131 (4.93)	71 (4.41)	0.35	0.92
low (%)	19 (0.86)	14 (0.69)	0.47	11 (0.41)	2 (0.12)	0.09	0.75
unknown (%)	235 (10.63)	156 (7.68)	<0.001	282 (10.61)	135 (8.39)	0.02	0.57

MMAS, Morisky Medication Adherence Scale.

$P_{interaction}$ for treatment randomization × medication burden status.

Table S11. Hazard Ratios for STEP Outcomes by Treatment Arm among Those with High and Low Medication Burden at Baseline (divided by baseline antihypertensive medication classes < 2 or ≥ 2)

Outcomes	<2 Medications			≥2 Medications			$P_{interaction}$
	Treatment		P Value or RR/HR (95% CI)	Treatment		P Value or RR/HR (95% CI)	
	Standard	Intensive		Standard	Intensive		
All participants, n	2659	2211		1609	2032		
Primary outcome (%)	107 (4.02)	66 (2.99)	0.74 (0.54-1.01)	89 (5.53)	81 (3.99)	0.73 (0.54-0.99)	0.99
All cause death (%)	38 (1.43)	40 (1.81)	1.34 (0.85-2.09)	26 (1.62)	27 (1.33)	0.82 (0.48-1.40)	0.19

RR, risk ratio; HR, hazard ratio.

$P_{interaction}$ for treatment randomization × medication burden status.

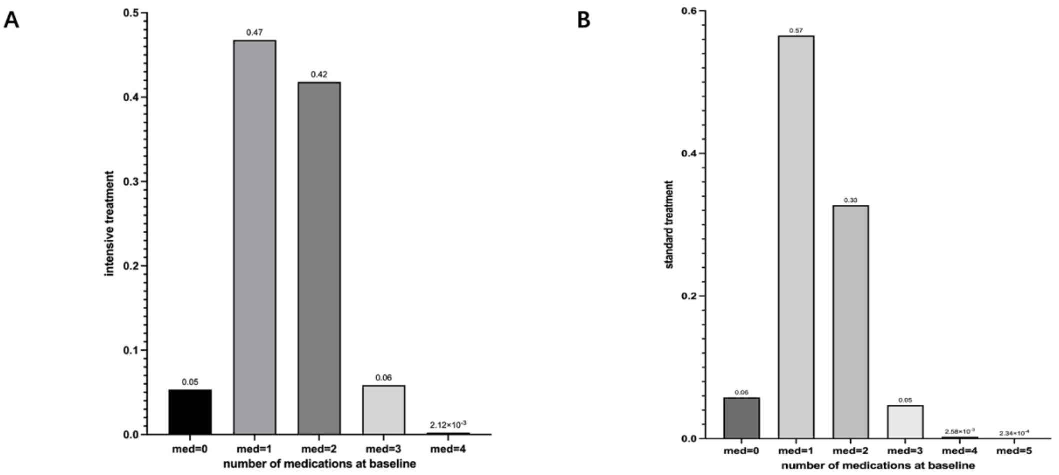

No. with Data					
Intensive treatment	226	1985	1774	249	9

No. with Data						
Standard treatment	246	2413	1397	200	11	1

Figure S1. Histogram of baseline medication number in STEP participants. A, Intensive treatment. B, Standard treatment.

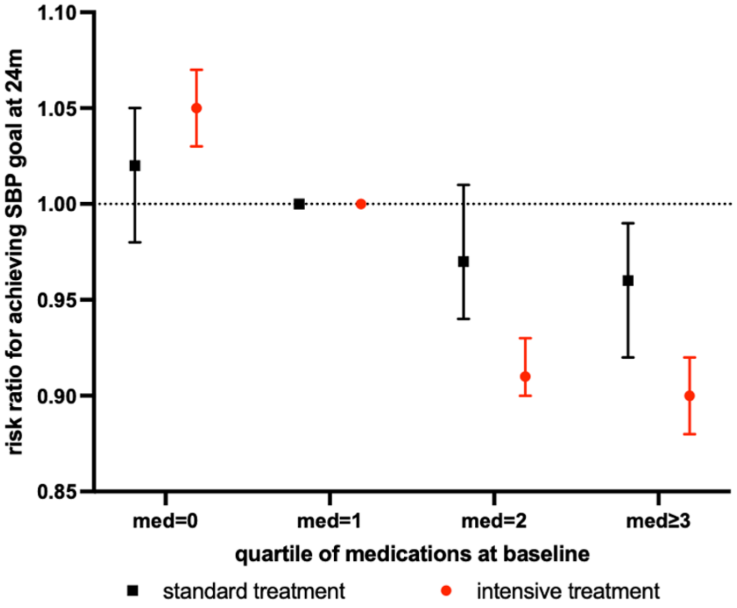

No. with Data				
Intensive treatment	193	1902	1725	252
Standard treatment	194	2315	1337	204

Figure S2. Relative risk ratios and 95% confidence intervals for achieving goals at 24-months post-randomization, by treatment group and baseline medication burden.

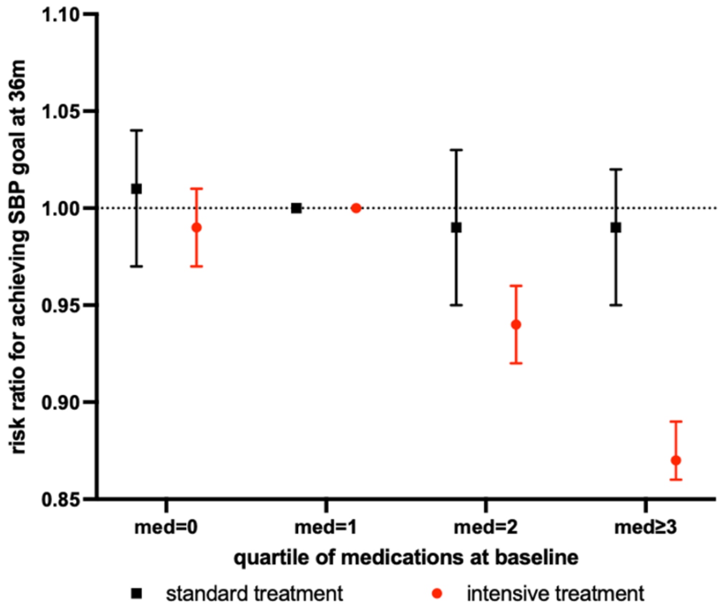

No. with Data				
Intensive treatment	176	1814	1641	226
Standard treatment	184	2233	1290	187

Figure S3. Relative risk ratios and 95% confidence intervals for achieving goals at 36-months post-randomization, by treatment group and baseline medication burden.

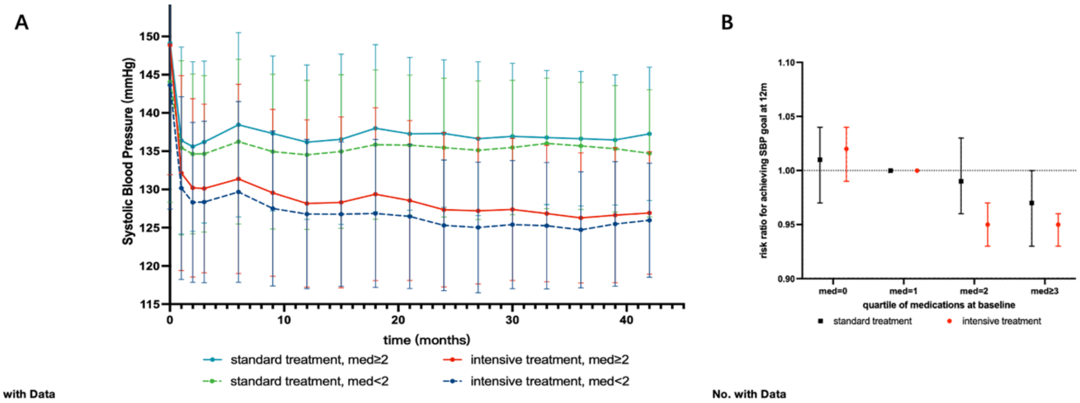

No. with Data								
Intensive treatment, <2 Meds	2211	2142	2109	2108	2095	2040	1990	881
Intensive treatment, ≥2 Meds	2032	1997	1977	1984	1977	1914	1867	1004
Standard treatment, <2 Meds	2659	2560	2523	2503	2509	2460	2417	1086
Standard treatment, ≥2 Meds	1609	1568	1563	1546	1541	1509	1477	764

Mean No. of Medications	0	6	12	18	24	30	36	42
Intensive treatment, <2 Meds	0.90	0.91	0.92	0.91	0.91	0.91	0.91	0.88
Intensive treatment, ≥2 Meds	2.13	2.13	2.13	2.13	2.13	2.12	2.12	2.14
Standard treatment, <2 Meds	0.91	0.92	0.93	0.92	0.92	0.92	0.92	0.91
Standard treatment, ≥2 Meds	2.14	2.14	2.14	2.14	2.14	2.14	2.14	2.17

No. with Data				
Intensive treatment	178	1931	1726	251
Standard treatment	187	2336	1358	205

Figure S4. Systolic blood pressure (SBP) and risk ratios for achieving the target SBP by treatment group and baseline medication burden. A, Mean SBP throughout the whole STEP trial (divided by baseline antihypertensive medication classes < 2 or ≥ 2). B, Adjusted risk ratios and 95% confidence intervals for achieving the target SBP at 12 months by quartile of baseline medication burden.

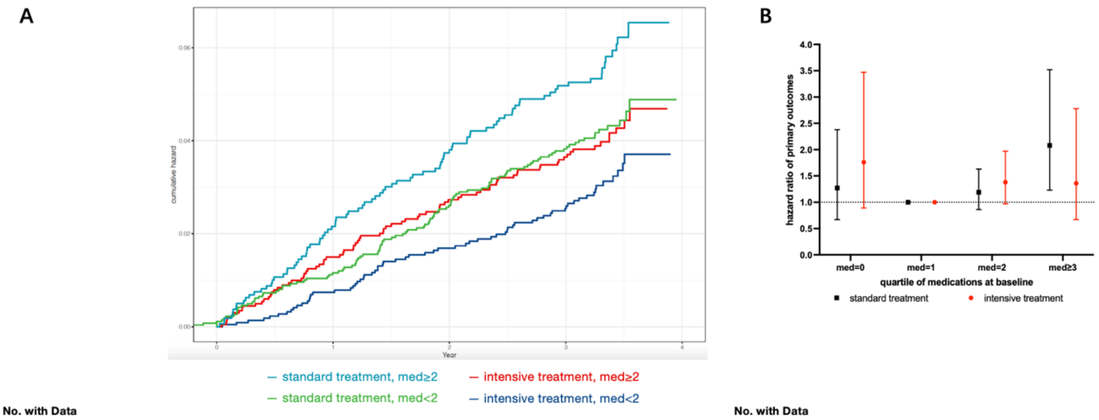

No. with Data					
Intensive treatment, <2 Meds	2211	2137	2056	1898	0
Intensive treatment, ≥2 Meds	2032	1972	1914	1795	0
Standard treatment, <2 Meds	2656	2526	2445	2264	0
Standard treatment, ≥2 Meds	1609	1543	1492	1400	0

No. with Data				
Intensive treatment	226	1985	1774	258
Standard treatment	246	2413	1397	212

Figure S5. Cumulative hazard plot and hazard ratios for primary outcomes by treatment group and baseline medication burden. A, Cumulative hazards for primary outcomes throughout the STEP trail (divided by baseline antihypertensive medication classes < 2 or ≥ 2). B, Adjusted risk ratios and 95% confidence intervals for experiencing primary outcomes at the end of the trial by quartile of baseline medication burden
